# Plasticity in the Structure of Visual Space

**DOI:** 10.1523/ENEURO.0080-17.2017

**Published:** 2017-06-23

**Authors:** Chen Song, Andrew M. Haun, Giulio Tononi

**Affiliations:** Department of Psychiatry, University of Wisconsin-Madison, Madison, WI 53719

**Keywords:** Lateral connections, visual plasticity, visual space

## Abstract

Visual space embodies all visual experiences, yet what determines the topographical structure of visual space remains unclear. Here we test a novel theoretical framework that proposes intrinsic lateral connections in the visual cortex as the mechanism underlying the structure of visual space. The framework suggests that the strength of lateral connections between neurons in the visual cortex shapes the experience of spatial relatedness between locations in the visual field. As such, an increase in lateral connection strength shall lead to an increase in perceived relatedness and a contraction in perceived distance. To test this framework through human psychophysics experiments, we used a Hebbian training protocol in which two-point stimuli were flashed in synchrony at separate locations in the visual field, to strengthen the lateral connections between two separate groups of neurons in the visual cortex. After training, participants experienced a contraction in perceived distance. Intriguingly, the perceptual contraction occurred not only between the two training locations that were linked directly by the changed connections, but also between the outward untrained locations that were linked indirectly through the changed connections. Moreover, the effect of training greatly decreased if the two training locations were too close together or too far apart and went beyond the extent of lateral connections. These findings suggest that a local change in the strength of lateral connections is sufficient to alter the topographical structure of visual space.

## Visual Abstract

**Figure:**
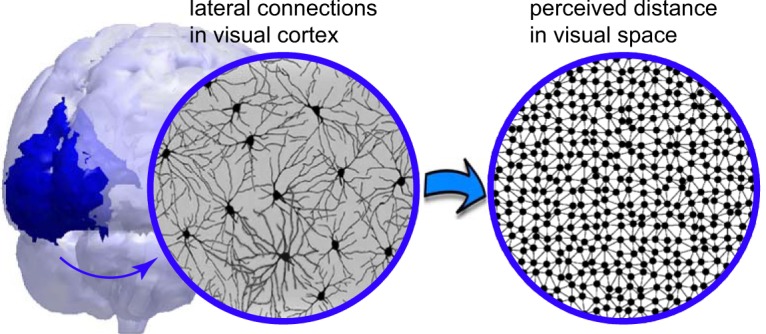

## Significance Statement

Given that visual space underlies visual perception, it is easy to take its topographical structure for granted. Indeed, most studies focus on object or feature perception that happens within visual space, without first considering the structure of visual space itself. Here we studied plasticity in the structure of visual space. We found that a local strengthening of lateral connections between retinotopically tuned visual cortical neurons, induced by synchronized, repetitive presentation of two-point stimuli, could lead to a contraction in perceived distance and a change in visual space structure. We propose lateral connections in the visual cortex as the mechanism that relates locations perceptually and shapes the structure of visual space.

## Introduction

More than a third of the human cerebral cortex is occupied by retinotopic maps of the visual field, in which individual neurons respond to specific locations in the visual field and nearby neurons to nearby locations (Sereno et al., 1995; Brewer and Barton, 2012; Katzner and Weigelt, 2013; Wang et al., 2014). This mapping between visual field and cortex can explain behavioral aspects of spatial localization (Rose, 1999). However, it is unclear what underlies the experience of spatial relations between locations: why do locations feel ordered in the specific way they do, apart from our abilities to behaviorally localize targets?

Whereas individual neurons in the visual cortex respond only to limited locations in the visual field, the lateral connections between these retinotopically tuned neurons instead allow distinct locations to be related. We hypothesize that the strength of lateral connections between neurons in the visual cortex determines the degree of perceived relatedness between locations in the visual field (Tononi, 2014; Tononi et al., 2016). This hypothesis explains how the organization of lateral connections, where the connection strength between neurons decays with their cortical separation (Clarke, 1994; Das and Gilbert, 1999), naturally gives rise to the topographical structure of visual space, where the perceived relatedness between locations decays with their visual field separation. Importantly, this hypothesis predicts that a change in the strength of lateral connections should alter the structure of visual space and affect the perceived relatedness between locations. Specifically, an increase in lateral connection strength should lead to an increase in perceived relatedness and a contraction in perceived distance. Moreover, the hypothesis predicts that the perceptual changes should occur not only between locations linked directly by the changed connections, but also between locations linked indirectly through the changed connections.

**Fig. 1.**
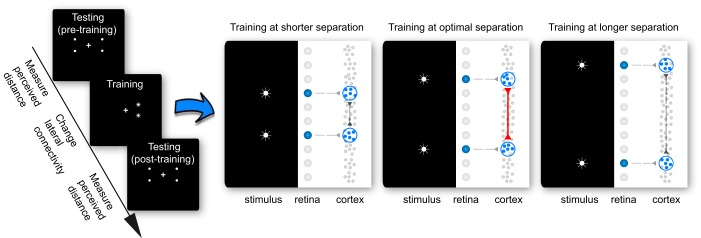
Experiment design. Each experiment run contained pretraining testing, training, and posttraining testing sessions. In the training session, we used synchronized, repetitive presentation of two-point stimuli to strengthen the lateral connections between two retinotopically tuned neuronal groups. Because the successful induction of synaptic plasticity requires the presence of direct connections between the two neuronal groups, there should be an optimal separation between the two-point stimuli for changing the lateral connection strength. At longer separation, the two neuronal groups would not be effectively connected; at shorter separation, the two neuronal groups could be partially overlapping; either way, the number of lateral connections involved and the net change in lateral connection strength would be less. In the testing session, we used a match-to-standard protocol to measure the perceived distance. Participants adjusted the physical separation of a dot pair in the untrained hemifield to match the perceived distance of a dot pair in the trained hemifield. The difference between the pre- and posttraining matches was taken to quantify the change in perceived distance and the effect of training.

To test this hypothesis, we used a Hebbian training protocol in which two-point stimuli were flashed in synchrony at separate locations in the visual field, to induce a short-term increase in the strength of lateral connections between two separate groups of neurons in the visual cortex (Yao and Dan, 2001; Fu et al., 2002, 2004; Caporale and Dan, 2008). A successful induction of synaptic plasticity requires the presence of direct connections between the two neuronal groups (Ganguly et al., 2000; Li et al., 2004). It follows that, depending on the distribution of lateral connection length, there should be an optimal separation between the two training locations at which the net increase in lateral connection strength is maximal and the contraction in perceived distance is accordingly maximal. At longer separation, the two neuronal groups would not be effectively connected; at shorter separation, the two neuronal groups could be partially overlapping; either way, the number of lateral connections involved and the net change in lateral connection strength would be less (Fig. 1). We therefore varied the separation between the two training locations from run to run and measured the effect of training on the perceived distance between the training locations and the perceived distance between the outward untrained locations.

## Materials and Methods

### Participants

Thirty healthy young adults (18 female) gave written informed consent to participate in the experiment that was approved by the Institutional Review Board of the University of Wisconsin-Madison. The experiments were conducted in a dark room with the display (ASUS PG278Q, 27-inch, 2560 × 1440 pixels, 120 Hz) providing the only significant source of light. Throughout the experiments, participants maintained central fixation and viewed the visual stimuli from a chin rest at a distance of 25 cm.

### Procedures

Each experiment run began with two testing sessions and was followed by three alternating cycles of training and testing sessions. In the training session, we used a Hebbian training protocol that was adapted from animal experiments and has been shown to strengthen the lateral connections in cat primary visual cortex (Yao and Dan, 2001; Fu et al., 2002, 2004; Caporale and Dan, 2008). To target lateral connections in human primary visual cortex (V1), we utilized the mirror-symmetry of human retinotopic organization. V1 is the only early visual cortical region whose ventral part (representation of upper visual field) and dorsal part (representation of lower visual field) are contiguous and are directly connected by lateral connections (Sereno et al., 1995; Brewer and Barton, 2012; Wang et al., 2014). In contrast, the ventral and dorsal parts of other early visual cortical regions (e.g., V2, V3) are segregated by V1 and may have a different organization of lateral connections. We therefore placed the two training locations in the upper and lower visual fields, to activate two separate groups of neurons in the ventral and dorsal parts of the visual cortex, respectively. Specifically, we flashed two synchronized, vertically separated point stimuli (5-pixel diameter) in the right hemifield at a rate of ON for 8.33 ms, OFF for 425 ms, and 275 cycles per training session (120 s). Participants were instructed to passively view the stimuli and minimize eye blinks.

In the testing session, we used a psychophysical match-to-standard protocol to measure the perceived distance between testing locations in the visual field. In each trial, two pairs of vertically separated dots (2-pixel diameter) were presented simultaneously for 200 ms in the two hemifields. Participants were instructed to report which dot pair appeared more separated or whether the two dot pairs appeared equally separated. The separation between the dot pair in the right hemifield was fixed at a standard value, and that in the left hemifield was adjusted by a one-up one-down double-interleaved staircase with a step size of 0.1 degrees. A total of 108 trials were obtained in two sessions of 54 trials each for the pretraining testing and three sessions of 36 trials each for the posttraining testing. The data were fitted with a logistic psychometric function to measure the point of subjective equality (PSE) where the two dot pairs appeared equally separated. The difference between the pre- and posttraining PSEs was taken to quantify the training-induced change in perceived distance.

Each participant took part in seven experiment runs. A single experiment run lasted 10 min and was followed by a 10-min compulsory rest to facilitate the recovery of pretraining baseline and minimize the accumulation of training effect across runs. In different experiment runs, we used training locations separated by 3.2, 3.6, 4.0, 4.4, 4.8, 5.2, or 5.6 degrees (one run each in random order) at a horizontal eccentricity of 6 degrees. The separations between testing locations were 0, 0.4, or 0.8 degrees larger than the separations between training locations. These visual field separations correspond to a cortical separation of 10–14 mm in human V1 (Schira et al., 2007) and overlap with the reported extent of lateral connections in primate visual cortex (Ringo, 1991; Burkhalter et al., 1993; Kaas, 2000; Levitt and Lund, 2002; Voges, et al., 2010; Ahmed et al., 2012; Lyon et al., 2014). Moreover, at an eccentricity of 6 degrees, the number of neurons activated by a point stimulus (cortical point image) should be minimal (Van Essen et al., 1984; Harvey and Dumoulin, 2011), thereby favoring the activation of distinct groups of neurons. The choices of training parameters were determined from pilot experiments, where we tested the effect of stimulus eccentricity (4, 6, 8 degrees), size (5-, 15-, 30-pixel diameter), pattern (two-point, two patches of dots, two checkboard wedges), and flashing profile (in synchrony or with interstimulus interval of 8.33, 16.67, 25.00, 33.33, 41.67, 50.00, 58.33, 66.67, 216.67 ms), through 276 experiment runs in three participants. Of the pilot parameters, those that favored focal activation of two separate groups of neurons (e.g., two-point as opposed to two patches of dots or two checkboard wedges) were used for formal experiments.

### Analysis

We quantified the training induced changes in the perceived distance between testing locations, and the dependence of training effects on the separation between training locations. We first performed repeated-measures ANOVA on the raw data, with pre-/posttraining and training separation as the within-subject factors. We then estimated how the training effect and its dependence on training separation differed across participants, presumably as a consequence of interindividual differences in visual cortical architecture (Andrews et al., 1997; Dougherty et al., 2003; Schwarzkopf et al., 2011, 2012; Song et al., 2011, 2013a, 2013b, 2015). For each participant, we fitted the training effect with a Gaussian function of the training separation and took the separation closest to the Gaussian peak as the optimal training separation. This procedure allowed us to calculate the group average after aligning each participant’s data to their optimal training separation.

## Results

After training, we observed a significant contraction in the perceived distance between the training locations, with the degree of contraction dependent on the training separation (repeated-measures ANOVA; effect of training on perceived distance: *F*(1,29) = 41.473, p < 10^−6^; quadratic trend in interaction between training effect and training separation: *F*(1,29) = 7.632, *p* = 0.010. The contraction was maximal at a training separation of 4.4 degrees, which corresponds to a cortical separation of 12 mm in human V1 (Schira et al., 2007) and falls within the extent of V1 lateral connections (Ringo, 1991; Burkhalter et al., 1993; Kaas, 2000; Levitt and Lund, 2002; Voges, et al., 2010; Ahmed et al., 2012; Lyon et al., 2014). Because of the mirror-symmetry of human retinotopic organization, V1 is the only early visual cortical region whose ventral part (representation of upper visual field) and dorsal part (representation of lower visual field) are contiguous, whereas the ventral and dorsal parts of other early visual cortical regions (e.g., V2, V3) are segregated by V1 (Sereno et al., 1995; Brewer and Barton, 2012; Wang et al., 2014). In these regions (e.g., V2, V3), a visual field separation of 4.4 degrees across horizontal midline would correspond to a cortical separation much larger than 12 mm and would fall outside the extent of lateral connections. The observation of a maximal training effect at a 4.4-degree visual field separation thus suggests the existence of an optimal separation at which the net increase in V1 lateral connection strength is maximal.

**Fig. 2.**
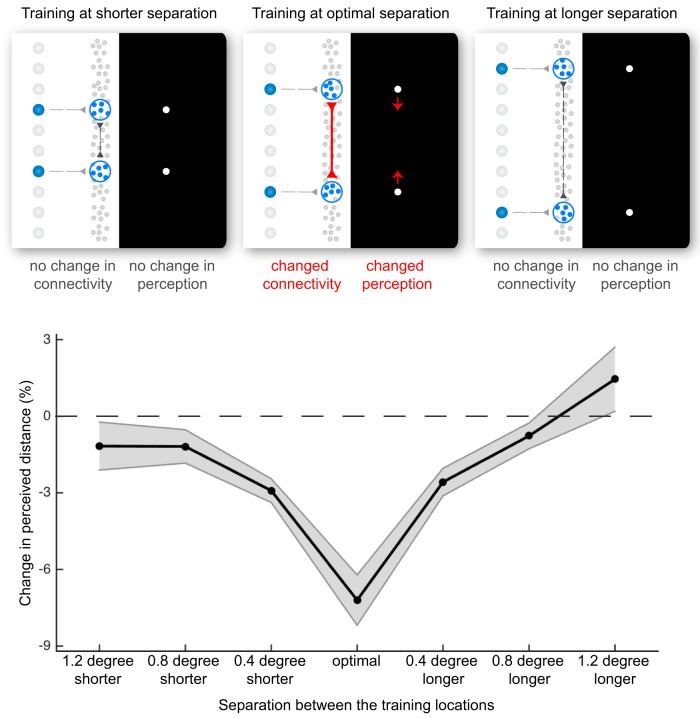
Change in perceived distance depends on training separation. The induction of synaptic plasticity and the change in lateral connection strength should be dependent on the separation between training locations. We measured the effect of training for a range of training separations. We observed a maximal contraction in perceived distance when training at a separation of 4.4 degrees. The contraction declined when training at shorter or longer separations. Black line, group average; shaded area, SEM (*n* = 30).

Because the surface area of V1 and the length of V1 lateral connections both exhibit considerable interindividual variability (Andrews et al., 1997; Dougherty et al., 2003; Schwarzkopf et al., 2011, 2012; Song et al., 2011, 2013a, 2013b, 2015), we expected the optimal training separation to vary from participant to participant. To account for the influence of this interindividual variability on the calculation of group average, we aligned each participant’s data to their optimal separation. The aligned group average (Fig. 2) revealed a 7.2% contraction of perceived distance when training at the optimal separation, which decreased to 2.6% when training at ±0.4 degrees from the optimal separation and 0.8% when training at ±0.8 degrees from the optimal separation. Similar results were obtained from the prealigned raw data, which revealed a group average of 3.5% contraction at the training separation of 4.4 degrees and a significant decrease to 1.1% when training at ±1.2 degrees away (as reflected by the significant quadratic trend in the ANOVA).

**Fig. 3.**
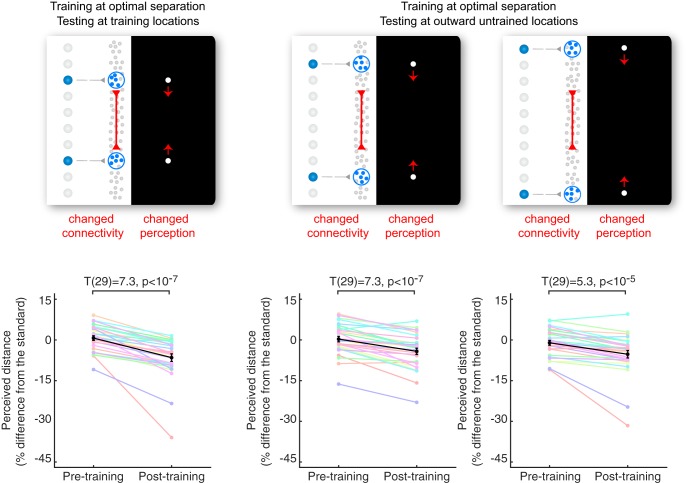
Change in perceived distance between untrained locations. A change in the strength of lateral connections should affect the perceived distance, not only between the training locations, but also between the untrained locations that span the training locations. We measured the effect of training for a range of testing locations. After training at the optimal separation, the perceived distance between the testing locations at 0.4 and 0.8 degrees outward from the training locations was significantly contracted. Black line, group average with SEM (*n* = 30); colored lines, individual participants. Paired-sample *t* tests are shown.

The hypothesis that lateral connections underlie the structure of visual space further suggests that the perceived distance should be changed not only between training locations, but also between outward untrained locations that are linked indirectly by the changed connections (Fig. 3). In line with this prediction, after training at the optimal separation, the perceived distance between the testing locations at 0.4 and 0.8 degrees outward from the training locations was contracted by a significant amount of 4.2% (*t*(29) = 7.3, *p* < 10^−7^) and 3.6% (*t*(29) = 5.3, *p* < 10^−5^), respectively. Moreover, the contraction (4.2%, 3.6%) produced by training at the optimal separation was even larger than the contraction (2.6%, 0.8%) produced by training at these testing locations, illustrating again that the effect of training is very weak at nonoptimal training separation.

## Discussion

Taken together, these findings suggest that a local strengthening of lateral connections induced by synchronized, repetitive presentation of two-point stimuli can lead to a contraction in perceived distance. More broadly, they suggest that lateral connections may underlie the topographical structure of visual space. The training protocol used in our study was adapted from animal studies and has been shown to strengthen lateral connection between neurons in cat V1 (Yao and Dan, 2001; Fu et al., 2002, 2004; Caporale and Dan, 2008). Because of the fine spatial scale over which the changes are expected to happen (∼10 mm) and the coarse spatial resolution of neuroimaging measures, it is difficult to ascertain the exact neural-level changes induced by the protocol in human participants. The protocol may have affected feedforward thalamocortical connections and changed receptive fields or response gains at the training locations (Kohler and Wallach, 1944; DeAngelis et al., 1995; Ganguly et al., 2000; Eyding et al., 2002; Hisakata et al., 2016). Similarly, we cannot rule out the training-induced changes in attention and feedback connections from fronto-parietal cortices to visual cortex (Anton-Erxleben et al., 2007; Klein et al., 2016).

Such feedforward or feedback mechanisms have been proposed to account for the perceptual changes induced by repetitive exposure to a visual stimulus and the transfer of perceptual effects across locations, features, or tasks (Fahle et al., 1995; Dill and Fahle, 1997; Goldstone, 1998; Sasaki et al., 2010; Zhang and Li, 2010). Usually, the transfer of perceptual effects to an untrained location is taken to indicate a feedback mechanism, whereas the dependence of perceptual effects on a retinotopic frame is taken to support a feedforward mechanism. However, neither feedforward nor feedback mechanisms can account for a U-shaped relation between the degree of perceptual changes and the separation of training locations, as was found here (Fig. 2). Moreover, a feedforward mechanism cannot explain the contraction in perceived distance between the untrained locations, as we also found (Fig. 3). Instead, our findings are exactly as predicted by the hypothesis that lateral connections linking neighboring neurons in the visual cortex shape the structure of experienced space (Fig. 1).

Given that visual space underlies visual perception, it is easy to take its topographical structure for granted. Indeed, most studies of visual perception focus on object or feature perception that happens within visual space, without considering the structure of space itself. One exception is a recent report of a paradoxical co-occurrence between decreased perceived density of dot textures and contracted perceived distance between dot pairs after adaptation to a large-field random dot stimulus (Hisakata et al., 2016). This finding cannot be explained by changes in neuronal response properties, and the authors instead proposed the scaling of an “internal metric” in the visual cortical system that relates distinct locations and specifies perceived distance (Hisakata et al., 2016). Our study suggests that this internal metric is provided by the organization and strength of lateral connections in retinotopic visual cortex.

Although the lateral connections are present throughout the visual cortex (Kaas, 2000; Levitt and Lund, 2002), different visual cortical regions may play different roles in the structure of visual space. Because of the cortical convergence, regions higher up in the visual hierarchy usually occupy less cortical area and have fewer neurons (Haug, 1987; Dougherty et al., 2003). Moreover, individual neurons in these visual cortical regions inherit the aggregate receptive fields of their multiple feedforward inputs (Sereno et al., 1995; Brewer and Barton, 2012; Katzner and Weigelt, 2013; Wang et al., 2014). By contrast, V1 at the bottom of the visual hierarchy has the largest cortical surface area, the largest number of neurons, and the smallest receptive fields (Andrews et al., 1997; Dougherty et al., 2003; Schwarzkopf et al., 2011, 2012; Song et al., 2011, 2013a, 2013b, 2015). The lateral connections in higher-up visual cortical regions will therefore span larger separations in the visual field and specify a coarser visual space, whereas the lateral connections in V1 will specify a finer visual space. These different spatial scales may jointly ensure a robust structure of visual space. Following on this proposal, an important next step would be to apply protocols that can target different visual cortical regions and selectively strengthen or weaken connections, such as theta burst transcranial magnetic stimulation (Huang et al., 2005; Salminen-Vaparanta et al., 2012; Rahnev et al., 2013; Silson et al., 2013), to examine the roles of individual visual cortical regions in the structure of visual space.

## Synthesis

Reviewing Editor: Li Li, New York University Shanghai Decisions are customarily a result of the Reviewing Editor and the peer reviewers coming together and discussing their recommendations until a consensus is reached. When revisions are invited, a fact-based synthesis statement explaining their decision and outlining what is needed to prepare a revision will be listed below. The following reviewer(s) agreed to reveal their identity: Bing Wu, Kevin Duffy.

Both reviewers think positively of this ms but required some non-major revisions and clarifications before the ms can be accepted for publication. I list the comments from the two reviewers below:

Reviewer 1:

The purpose of this study was to test a hypothesis derived from the integrated information theory: “lateral connections linking neighboring neurons in visual cortex shape the structure of experienced space”. In a Hebbian training experiment, the authors asked their subjects to passively view a pair of flashing dots for 120 seconds and then to perform a dot-separation-comparison task in the post-training test. The results showed that the dot-separation was under-perceived after the training, presumably due to enhanced lateral neural connections.

Overall, it is very good work: The experiment was well designed and properly conducted, the data analysis was adequate. And the manuscript is well written and easy to read. I also think this work is of high interest to readers of this e-Journal. While such a demonstration of a link between neural plasticity and behavioral consequence is important and noteworthy, I do have some concerns and comments, as discussed below.

(1). My first question is, where does such enhancement of lateral connections occur? The authors simply attribute the behavioral effects to “lateral connections linking neighboring neurons in visual cortex”. This statement sounds too vague particularly for readers in visual neuroscience. Moreover, the authors showed the training effects were maximal at a training separation of 4.4 degrees, and “this optimal separation corresponds to the upper extent of lateral connections in visual cortices (Ahmed et al., 2012; Lyon et al., 2014) ”(Line 106-107). The two cited work measured the long-range lateral connections in V1 (Lyon et al., 2014) and V5 (Ahmed et al., 2012), respectively, in macaque monkeys. How were the work related to the findings observed here in humans?

(2) Several models have been proposed for explain perceptual learning, I suggest the authors to significantly expand the Discussion section to discuss their findings in the context of not only the integrated information theory but also other alternative explanations.

Reviewer 2:

This manuscript, “Plasticity in the structure of visual space”, explores the influence that psychophysical training has on distance perception. Results are interpreted in the context of a training regime being capable of altering intrinsic lateral connections in visual cortex and thereby modifying distance perception. This is an interesting study that has the potential of contributing important insight into our understanding of the mechanisms that underlie the topographical structure of visual space. Below is a collection of comments and questions the authors can consider before the manuscript is published.

Stated in the Introduction, the objective of this study is to use psychophysical training as a means of targeting and modifying lateral connections between neurons in visual cortex. What is the evidence that training actually strengthens lateral connections in isolation of myriad other possible neural changes produced by the selected training regime? Also, what is the reasoning behind assuming that the meaningful neural changes produced by training would be specific to visual cortex and not antecedent brain areas?

The authors specifically predict that increased lateral connectivity, which I presume means increased synaptic strength, should lead to a decrease in perceived distance. Why would strengthening interconnections necessarily lead to decreased perceived distance rather than increased perceived distance? What is it about an increase in synaptic strength that would yield a perceptual shift in this particular direction? In the absence of consideration of the somewhat vague concept of relatedness, why does the increase in connectivity not lead to a decrease in perceptual distance? Would a targeted decrease in connectivity be expected to produce an increase in perceptual distance?

Although this study operates under the theoretical view that intrinsic lateral connections underlie the structure of visual space (stated throughout the manuscript), axonal guidance mechanisms that organize retinal output in the first place is not acknowledged as a possible source underlying such structure. It is upon this retinotopic map that lateral connections are able to subsequently modify the perception of visual space. Organized retinal input would seem to be the principal substrate and perhaps should be acknowledged.

In the last paragraph of the Introduction the authors state that for successful induction of synaptic plasticity (increased strength, I presume), there should be an optimal separation between the two point stimuli - with short separation decreasing the number of lateral connections involved. It is not clear from the text why the authors expect that shorter separation would engage fewer lateral connections. Is this because small dot separation would fail to elicit activity from two distinct neuron populations that would not be subject to lateral connection? Or is this logic instead related to known measurements of lateral connectivity relative to visuotopic mapping?

At higher visual areas, receptive fields are much larger, presumably in part due to convergence of retinotopically organized input. In these visual areas lateral connections would be expected to link much larger distances than lower visual areas like V1. How do the current results reconcile the difference in receptive field size across visual areas? Is the contraction of perceived distance possibly mediated exclusively by neurons of V1? Perhaps this is reason to think it is.
